# Temporal Changes in Affective Symptoms, Headache Burden, and Quality of Life Following rTMS Treatment in Migraine: A Longitudinal Study

**DOI:** 10.3390/healthcare14091242

**Published:** 2026-05-04

**Authors:** Robert Zgarbura, Alexandru Pavel, Oana-Andreea Parliteanu, Jari Sabri, Catalina Tudose

**Affiliations:** 1 Psychiatry Department, University of Medicine and Pharmacy “Carol Davila”, 041914 Bucharest, Romania; robertconstantinzgarbura@gmail.com (R.Z.); catalina.tudose52@gmail.com (C.T.); 2 FutureMeds, 031441 Bucharest, Romania; 3 PAX Clinic, 030167 Bucharest, Romania; 4 Diabetes Department, Pneumology Institute “Marius Nasta”, 050159 Bucharest, Romania; 5 Faculty of Medicine, University of Medicine and Pharmacy “Titu Maiorescu”, 031593 Bucharest, Romania; 6 Faculty of Physical Education and Sports, “Spiru Haret” University, 041906 Bucharest, Romania; jari.sabri@spiruharet.ro

**Keywords:** rTMS, migraine, depression, anxiety, quality of life, neuromodulation, theta burst stimulation, headache disability, affective symptoms, longitudinal study

## Abstract

**Highlights:**

**What are the main findings?**

- rTMS was associated with improvements in anxiety, depression, and migraine-related quality of life. Distinct temporal trajectories were observed across affective and functional domains.

**What is the implication of the main finding?**

- Headache burden improved early, while affective symptoms and quality of life continued improving at follow-up.
- Longitudinal assessment revealed delayed benefits not captured by post-treatment endpoints alone.
- Findings should be interpreted cautiously given the one-arm design.

**Abstract:**

Background: Migraine is frequently associated with anxiety, depression, and reduced quality of life, contributing to substantial functional impairment. Objective: To examine longitudinal changes in affective symptoms, headache-related burden, and quality of life following repetitive transcranial magnetic stimulation (rTMS) in individuals with migraine. Methods: In this one-arm longitudinal study, 32 adults with migraine underwent 10 sessions of rTMS. Anxiety (HAMA), depression (HAMD), and migraine-specific quality of life were assessed at baseline, post-treatment, and 3-month follow-up, while headache impact (HIT-6) and disability (MIDAS) were evaluated at baseline and post-treatment. Repeated-measures analyses and paired comparisons were conducted. Results: Significant improvements over time were observed for anxiety, depression, and quality of life (all *p* < 0.001). Anxiety showed progressive improvement through follow-up, while depressive symptoms improved early with further consolidation at 3 months. Migraine-related quality of life increased significantly across all timepoints. Headache impact and disability decreased significantly following treatment (both *p* < 0.001), with large effect sizes. Conclusions: rTMS was associated with improvements in affective symptoms, migraine-related burden, and quality of life. However, given the one-arm design, these findings should be interpreted cautiously. Controlled studies are needed to confirm these results.

## 1. Introduction

Migraine is a highly prevalent and disabling neurobiological disorder characterized by recurrent headache attacks and substantial cognitive, emotional, and functional impairment [1,2]. Beyond pain, migraine is now conceptualized as a disorder of network dysfunction, involving aberrant excitability and altered connectivity across cortical and subcortical circuits implicated in sensory processing, emotional regulation, and executive control [3,4,5]. These alterations could explain the frequent co-occurrence of migraine with anxiety and depressive symptoms, as well as the impact on quality of life [6,7].

These neurobiological alterations likely underlie the high prevalence of psychiatric comorbidity in migraine—present in up to 50% of individuals—with anxiety and depressive disorders being the most common [7,8]. This overlap reflects shared biological mechanisms, including dysfunction in fronto-limbic circuits, altered serotonergic and dopaminergic signaling, and maladaptive changes within the salience and default mode networks, rather than a mere psychological reaction to chronic pain [3,4,9,10]. Critically, affective symptoms in migraine are associated with greater headache frequency, increased disability, poorer treatment response, and reduced quality of life, suggesting a bidirectional [7,11] and self-reinforcing relationship between pain and emotional dysregulation [6,12,13,14].

Repetitive transcranial magnetic stimulation (rTMS) is a non-invasive neuromodulatory intervention that modulates cortical excitability and network plasticity [15,16]. By delivering patterned magnetic pulses to targeted cortical regions—most commonly the dorsolateral prefrontal cortex (DLPFC)—rTMS engages large-scale brain networks implicated in both pain processing and affect regulation, positioning it as a mechanistically relevant treatment for migraine and its psychiatric comorbidities [17,18,19]. There is substantial evidence supporting the efficacy of rTMS in major depressive disorder [20] and in pain disorders [21,22,23], including migraine. In migraine populations, a meta-analysis [24] of eight randomized controlled trials (RCTs; n = 361) demonstrated that rTMS reduced migraine attack frequency and headache intensity compared to sham, with significant improvements in disability and a favorable adverse event profile [21,22,23]. In depression, a meta-analysis [25] of 23 RCTs (n = 960) confirmed superiority of active rTMS over sham for both response and remission rates. Similar benefits have been reported in anxiety disorders, though with greater heterogeneity across studies [26,27].

Despite this evidence, most studies have evaluated single-domain endpoints—typically headache frequency or depressive symptoms—without characterizing the temporal evolution of affective symptoms, disability, and quality of life within the same cohort [16,17]. This is a clinically relevant gap: neuromodulatory effects may emerge at different rates across symptom clusters, reflecting distinct underlying neural mechanisms; early improvements in headache burden may precede and facilitate later improvements in affective functioning and quality of life; and longitudinal assessment captures delayed or cumulative neuroplastic effects that post-treatment endpoints alone cannot detect. To our knowledge, no study has examined the simultaneous trajectory of anxiety, depression, migraine-related disability, and quality of life within a unified longitudinal framework following rTMS.

The present study aimed to investigate longitudinal changes in affective symptoms, migraine-related burden, and quality of life following a course of rTMS in individuals with migraine.

Therefore, the present study aimed to characterize the temporal evolution of these domains following rTMS treatment in individuals with migraine, with particular focus on differential response trajectories across clinically distinct outcome domains. Based on existing evidence, we formulated the following hypotheses: (1) rTMS would produce significant reductions in anxiety symptoms (HAMA) from baseline to post-treatment, with effects maintained or further consolidated at 3-month follow-up, consistent with evidence of rTMS efficacy in anxiety disorders; (2) rTMS would similarly reduce depressive symptoms (HAMD), given the robust body of evidence supporting rTMS in major depressive disorder; (3) migraine-specific quality of life (Migraine-QoL) would improve significantly over time, reflecting the cumulative functional benefits of reduced headache burden and improved affective state; (4) headache impact (HIT-6) and disability (MIDAS) would decrease significantly following treatment, in line with meta-analytic evidence of rTMS reducing migraine frequency and disability. Exploratorily, we anticipated that improvements in headache burden might precede improvements in affective symptoms and quality of life, consistent with a cascade model of clinical response.

## 2. Materials and Methods

### 2.1. Study Design and Participants

This study had a one-arm, longitudinal design. Given the real-world clinical context of this study and its exploratory objectives, a sham-controlled design was not feasible; the one-arm design was considered appropriate for an initial characterization of temporal response trajectories in a routine clinical setting. Between October 2023 and December 2025, we enrolled participants diagnosed with migraine according to ICHD Guidelines [2] who presented at a local neurology clinic for rTMS sessions and prospectively collected sociodemographic and clinical data. Inclusion criteria were: (1) confirmed diagnosis of migraine according to ICHD-3 criteria; (2) age 18 years or older; (3) ability to provide written informed consent. Exclusion criteria were: (1) contraindications to rTMS (history of epilepsy, intracranial metallic implants, pacemaker); (2) active psychotic disorder or current manic episode. All procedures were conducted in a specialized outpatient neurology clinic providing rTMS treatment. Participants were enrolled consecutively, reflecting routine clinical practice. No participants discontinued the intervention once initiated. All participants completed baseline and post-treatment assessments, and follow-up data at 3 months were available for all included individuals.

A licensed psychiatrist applied the Hamilton Anxiety Scale (HAMA) [28], Hamilton Depression Scale (HAMD) [29], and Migraine Quality of Life (Migraine-QoL) [30] at baseline, after 10 sessions and 3 months after baseline. The same rater applied Headache Impact Test (HIT-6) [31] and Migraine Disability Assessment Test (MIDAS) [32] at baseline and after 10 sessions. Assessor was not blinded to the timing of evaluation. However, the use of the same rater across all timepoints ensured consistency in assessments and reduced inter-rater variability. A different, trained clinician conducted the rTMS sessions.

Treatment stability was verified through clinical documentation at each assessment visit, confirming that no changes in medication type, dose, or psychological treatment status occurred for any participant during the study period.

The protocol and observational study were not preregistered. The overall design of the paper was structured following the STROBE checklist (Strengthening the Reporting of Observational Studies in Epidemiology) (See Supplementary material).

### 2.2. Measures

HAMA is a clinician-rated instrument comprising 14 items [28]. Each item is scored from 0 (absence of symptoms) to 4 (severe symptoms), yielding a total score between 0 and 56, with higher scores indicating greater anxiety severity. The original English version was used since it is a clinician-rated scale. Cronbach’s alpha for HAMA at baseline was 0.642, slightly lower than reported in the literature [33]. This could be explained by the small sample size and migraine diagnosis, which tends to create higher variability compared to studies where the population included is focused on anxiety disorders, thus diluting the discriminant structure of pure anxiety items [34,35].

HAMD is a clinician-administered 17-item scale in which higher total scores reflect more severe depression [36]. Cronbach’s alpha for HAMD at baseline was 0.786, consistent with literature data [37].

The original English version was used. Both HAMA and HAMD are originally in English and are widely used internationally in their original form in clinical settings.

Migraine-related quality of life was measured using Migraine-QoL, a 13-item instrument designed for individuals with migraine. The scale includes three domains—Role Restrictive, Role Preventive, and Emotional Functioning—and higher scores denote better perceived quality of life. The Migraine-QoL was administered in its original English version, as no validated Romanian translation is currently available. Given that this is a clinician-assisted administration, patients were supported in understanding item content as needed. Cronbach’s alpha was 0.894, consistent with literature data [38].

Headache impact was assessed with the Headache Impact Test (HIT-6), a self-report questionnaire consisting of six items evaluating headache severity and its effects on daily activities, including social and occupational functioning, concentration, vitality, and psychological distress. Higher scores represent higher headache severity. The Romanian version was used [39,40]. Cronbach’s alpha was situated at 0.811 with similar ranges described in the literature [39].

Migraine-related disability was evaluated using the Migraine Disability Assessment (MIDAS) questionnaire, a brief self-administered measure that captures headache-related disability over the preceding three months by assessing days of missed or reduced activity at work, at school, and during leisure time, with higher scores indicating greater disability. The Romanian translation was used [41]. Cronbach’s alpha was 0.640, slightly lower than values described in the literature [41], but consistent with data showing increased variability among clinical samples compared to the original validation paper and the possible impact of occupational status on item scoring [42,43].

HIT-6 and MIDAS were assessed at baseline and post-treatment only. MIDAS evaluates disability over a 3-month recall period, which overlaps with the follow-up interval; therefore, it was not re-administered at 3 months to avoid redundancy and recall bias. Similarly, HIT-6 was used to capture short-term changes in headache impact following the intervention.

### 2.3. rTMS Protocol

Participants underwent 10 sessions of rTMS using a modified intermittent theta burst stimulation (iTBS) paradigm. Whereas standard iTBS consists of bursts of 3 pulses at 50 Hz repeated at 5 Hz, the present protocol delivered trains of 20 pulses at 10 Hz, organized in 2 s stimulation periods with 8 s inter-train intervals, for a total of 20 trains and 400 pulses per session. This modification reflects the clinical protocol routinely employed at the treatment site and is acknowledged as a deviation from canonical iTBS parameters.

Stimulation targeted the dorsolateral prefrontal cortex (DLPFC) and was delivered using a MagVenture A/S, Farum, Denmark model MagPro R20 with a figure-of-eight coil. Motor threshold was determined prior to treatment, and stimulation intensity was set at 120% of the motor threshold.

rTMS sessions were typically administered once daily on consecutive weekdays; however, minor variations in scheduling occurred depending on patient availability.

The present study obtained approval from the local ethics committee. All participants signed informed consent before inclusion. This study was conducted in accordance with the Declaration of Helsinki [44].

### 2.4. Statistical Analysis

All analyses were performed using Statistical Package for Social Sciences (SPSS) version 26 (IBM Corp, 2019). Descriptive statistics were used to characterize the sample. The normality of the difference scores for paired comparisons (HIT-6 and MIDAS) was assessed using the Shapiro–Wilk test. HIT-6 did not significantly deviate from normality, while MIDAS showed a mild deviation (*p* = 0.034). Given the sample size (n = 32), paired *t*-tests were considered robust to moderate violations of normality.

For outcomes measured at three time points (HAMA, HAMD, Migraine-QoL), repeated-measures analysis of variance (RM-ANOVA) was used to evaluate changes over time. Mauchly’s test of sphericity was examined, and Greenhouse–Geisser corrections were applied where appropriate. Effect sizes were reported as partial eta squared (η^2^p). As commonly described, the η^2^p ranges from 0 to 1, with the following interpretations: 0.01 (small), 0.06 (medium), and 0.14+ (large). Given the exploratory longitudinal design and consecutive recruitment strategy, no a priori sample size calculation was performed before study initiation. However, to assess sample adequacy, we conducted a G*Power 3.1 estimation for a repeated-measures ANOVA (within-subject factor, 3 timepoints), using a medium effect size (*f* = 0.25), α = 0.05, and power = 0.80. The estimated minimum required sample was 28 participants; thus, the included sample of 32 participants was considered adequate.

Given the exploratory design and the limited sample size, no formal correction for multiple comparisons was applied as the primary analysis strategy, to avoid inflating Type II error. As a sensitivity check, Bonferroni correction was applied to pairwise comparisons within each RM-ANOVA (adjusted alpha = 0.017 for three comparisons per outcome); all comparisons remained statistically significant following correction.

For HIT-6 and MIDAS, which were measured at two time points only, paired-samples *t*-tests were conducted. Effect sizes for paired comparisons were calculated using Cohen’s *d* for dependent samples, defined as the mean change divided by the standard deviation of the change scores (i.e., within-subject differences). This approach differs from the pooled standard deviation method used in independent-group designs.

Common interpretations for these effect sizes are: small (0.2), medium (0.5) and large (0.8). All tests were two-tailed, with a significance threshold of *p* < 0.05.

**Figure 1:**
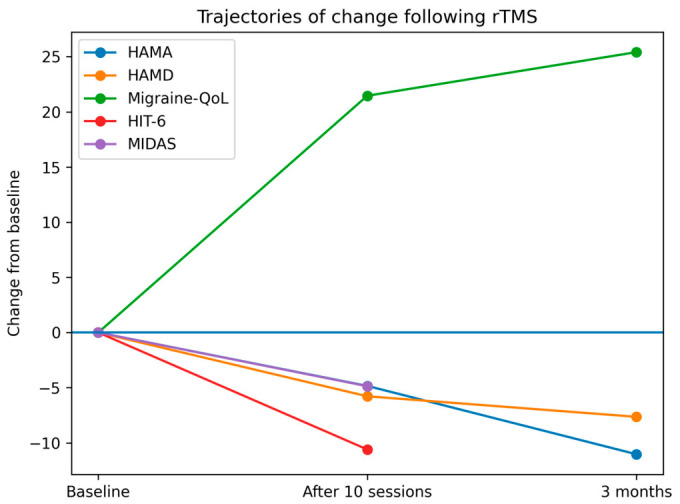
Trajectories of change in anxiety (HAMA), depression (HAMD), migraine-related quality of life (Migraine-QoL), headache impact (HIT-6), and migraine disability (MIDAS) following rTMS. Values represent mean change from baseline. Negative values indicate symptom improvement for HAMA, HAMD, HIT-6, and MIDAS, while positive values indicate improvement in Migraine-QoL.

Figure 1 was generated using an AI-assisted tool (ChatGPT) and subsequently verified and approved by all authors.

## 3. Results

The whole sample included 32 participants. Overall, 71.9% were female, middle-aged (mean age 41.88 years), and most were receiving stable pharmacological treatment at study entry. No changes in concomitant treatments were made during the study period. The entire sample characteristics are presented in Table 1. The evolution of the outcome measures across all timepoints (as mean scores and standard deviations) are presented in Table 2.

### 3.1. Changes over Time

Mauchly’s test of sphericity indicated that the sphericity assumption was met for HAMA (W = 0.926, *p* = 0.317); uncorrected results are therefore reported. Sphericity was violated for HAMD (W = 0.597, *p* = 0.001, ε = 0.713) and Migraine-QoL (W = 0.463, *p* < 0.001, ε = 0.651); Greenhouse–Geisser corrected values are reported for these outcomes.

All three outcomes measured across three timepoints showed significant main effects of time (all pairwise comparisons significant, *p* ≤ 0.006 after Bonferroni correction; Table 3). Anxiety symptoms demonstrated a progressive and sustained reduction, with the largest improvement observed between baseline and 3-month follow-up. Depressive symptoms showed a marked early response following treatment completion, with further consolidation at follow-up. Migraine-specific quality of life improved significantly across all timepoints, with continued gains at 3 months. Detailed mean scores, pairwise comparisons, and effect sizes are presented in Table 3 and Table 4, and trajectories are illustrated in Figure 1.

Depressive symptoms also showed a significant effect of time (*p* < 0.001, η^2^p = 0.764), though slightly reduced compared to anxiety. Paired comparisons indicated significant reductions from baseline to post-treatment with further improvement at three-month follow-up (all *p* ≤ 0.002). Migraine-QoL scores improved significantly over time (*p* < 0.001, η^2^p = 0.752). Paired analyses demonstrated significant improvement across all three timepoints (all *p* ≤ 0.001). Evolution of symptom scores is also presented in Figure 1.

### 3.2. Migraine Disability and Impact

Both headache impact (HIT-6) and disability (MIDAS) decreased significantly from baseline to post-treatment (both *p* < 0.001), with large effect sizes (Table 4).

## 4. Discussion

The present study examined longitudinal changes in affective symptoms, migraine-related burden, and quality of life following rTMS, revealing distinct but converging trajectories of improvement across domains. Given the one-arm observational design, the changes observed following rTMS reflect temporal associations rather than established causal effects, and all mechanistic interpretations should be considered hypothesis-generating. Even though the changes did not reflect a uniform pattern, there was a time-dependent evolution of response, with early symptom improvement followed by sustained or progressive gains at follow-up.

Anxiety symptoms, as measured by HAMA, showed a sustained reduction over time, with significant improvement observed both after the intervention and at the three-month follow-up. These findings suggest that the observed reductions in anxiety were not limited to the acute intervention period. This trajectory is consistent with the possibility of delayed or cumulative effects on anxiety, as well as indirect benefits from reduced headache burden—though these interpretations remain speculative in the absence of a control condition. This pattern is broadly consistent with previous findings; however, existing studies show considerable heterogeneity in response trajectories, likely reflecting differences in stimulation protocols, target regions, and patient populations. Moreover, most prior studies have focused on single-domain outcomes, limiting direct comparability with the present multidimensional longitudinal approach [45,46,47].

Depressive symptoms demonstrated a rather different pattern. HAMD scores showed a large early response, substantially improving after 10 treatment sessions, followed by additional but smaller gains at follow-up. This trajectory suggests a model of early symptom response with subsequent consolidation, rather than continuous linear improvement. Such a pattern is consistent with some previous observations [48,49]; however, other studies have reported more variable or gradual response trajectories, suggesting that symptom evolution may depend on baseline severity, stimulation parameters, and concurrent treatments.

Migraine-specific quality of life improved significantly across all time points, with marked gains present after treatment completion and further improvement at three months. The magnitude of QoL improvement continued to increase at follow-up, suggesting that functional recovery may lag symptomatic relief. This finding supports the notion that improvements in daily functioning and emotional well-being may require time to translate into meaningful quality-of-life changes, even after core symptoms begin to improve [50,51].

Reductions in migraine-related burden and disability (assessed using HIT-6 and MIDAS) were evident early, with significant improvements observed immediately after treatment. Given that these measures capture headache impact and activity limitation, the early response suggests that improvements in migraine-related functional measures may emerge rapidly following the intervention, though the absence of a control group precludes causal attribution. Given the study design, it is difficult to say if there is a causality relation, meaning whether improvements in headache burden preceded or contributed to changes in affective symptoms, or vice versa, cannot be established from the present design and warrants investigation in future controlled studies.

The observed temporal patterns may be consistent with a potential cascade-like relationship, in which early reductions in headache impact are followed by improvements in affective symptoms and, ultimately, in quality of life. However, this interpretation remains speculative and cannot be formally tested within the present dataset. These temporal differences emphasize the importance of longitudinal assessment when evaluating rTMS outcomes in migraine populations, as short-term endpoints may underestimate the full extent of clinical benefit. Most of the data that we have found focused on trajectories of symptoms in depression (some also including anxiety) [52,53,54,55]. To our knowledge, few studies have examined the simultaneous trajectories of depression, anxiety, migraine burden, and quality of life within the same participant sample.

Our results highlight the value of considering domain-specific trajectories rather than relying on single timepoint outcomes. The sustained and, in some cases, delayed improvements observed at follow-up suggest that rTMS effects may continue to evolve after treatment completion. Future studies with larger samples and controlled designs should further investigate predictors of these trajectories and clarify the mechanisms underlying delayed functional recovery.

### 4.1. Strengths

The present study has several strengths. First, it employed a longitudinal design with repeated assessments across multiple timepoints, allowing for the characterization of temporal trajectories of response rather than relying solely on pre–post comparisons. This approach provides a more nuanced understanding of how different symptom domains evolve following rTMS. Second, this study simultaneously evaluated multiple clinically relevant domains, including anxiety, depressive symptoms, migraine-related disability, and quality of life, within the same participant sample. This integrative approach allows for a more comprehensive assessment of treatment effects and supports the exploration of interrelated changes across domains. Third, the inclusion of a 3-month follow-up enabled the identification of delayed and sustained effects, which are often not captured in studies limited to immediate post-treatment outcomes. Fourth, participants were recruited consecutively from a real-world clinical setting, enhancing the ecological validity and clinical applicability of the findings. Finally, the use of standardized and widely validated clinical instruments, along with consistent assessment by the same trained rater, supports the reliability of the measurements and reduces inter-rater variability.

### 4.2. Limitations

While our results provide valuable insights into the temporal dynamics of rTMS effects in migraine, several limitations should be acknowledged. First, the sample size was relatively small, reflecting the exploratory nature of the study, and may limit the generalizability of the findings. Although effect sizes were large, variability within a small sample may influence the stability of estimates. Second, this study employed a one-arm, non-randomized longitudinal design without a sham-controlled comparison group. As a result, placebo effects, regression to the mean, and natural fluctuations in migraine symptoms cannot be excluded. Consequently, the observed improvements reflect temporal associations and do not allow causal inferences regarding the effects of rTMS. Third, the naturalistic design introduces the potential for selection bias, as participants were recruited consecutively from a clinical setting and may not be representative of the broader migraine population. Fourth, participant heterogeneity represents an important limitation. A substantial proportion of participants were receiving concurrent psychiatric treatments, including antidepressants, antipsychotics, and psychotherapy, all of which may independently influence affective outcomes. Although treatment stability was maintained during the study period, the contribution of these interventions cannot be disentangled from the effects of rTMS. Fifth, no formal adjustment for potential confounding variables was performed, and therefore, residual confounding cannot be excluded. Sixth, the use of clinician-rated and self-report instruments introduces potential measurement bias, including expectancy effects and rater-dependent variability, particularly given the lack of assessor blinding. Seventh, headache-related measures (HIT-6 and MIDAS) were not assessed at the 3-month follow-up, limiting the ability to fully characterize longitudinal relationships between headache burden and affective outcomes. Eighth, the follow-up period was limited to three months, restricting conclusions regarding long-term durability of treatment effects. Ninth, the study was conducted in a single clinical center, which may limit external validity and generalizability to other populations or healthcare settings. Finally, the stimulation protocol differed from canonical iTBS parameters, which may limit comparability with other studies using standard protocols.

### 4.3. Future Directions

Future research should aim to build on these findings through larger, adequately powered studies employing randomized, sham-controlled designs to better isolate the specific effects of rTMS from placebo-related responses. Longitudinal studies with extended follow-up periods are needed to determine the durability of treatment effects and to assess whether maintenance rTMS protocols may be beneficial in sustaining clinical improvements. Further investigation into predictors of response, including clinical, demographic, and neurobiological factors, may help identify subgroups of patients most likely to benefit from rTMS and support more personalized treatment approaches. Additionally, future studies integrating neuroimaging or electrophysiological measures could help clarify the mechanisms underlying the observed temporal trajectories, particularly the relationship between early changes in headache burden and subsequent improvements in affective symptoms and quality of life.

Finally, the development and validation of culturally adapted instruments, including migraine-specific quality-of-life measures, would enhance the accuracy and generalizability of findings in diverse populations.

## 5. Conclusions

In this exploratory longitudinal study, rTMS was associated with improvements in affective symptoms, migraine-related burden, and quality of life, with distinct temporal patterns across domains. These findings highlight the importance of longitudinal assessment in migraine treatment. However, controlled studies are required to confirm these results and clarify underlying mechanisms.

## Figures and Tables

**Table 1:** Social, demographic and clinical characteristics of the sample.

	rTMS Participants (N = 32)
Gender	
Male	9 (28.1%)
Female	23 (71.9%)
Age	41.88 (15.94)
Urban area	28 (87.5%)
Rural area	4 (12.5%)
Education	
High school	15 (46.9%)
University	17 (53.1%)
Occupational status	
Unemployed	9 (28.1%)
Employed	23 (71.9%)
In a relationship	18 (56.3%)
Single	14 (43.7%)
Number of previous rTMS trials	1.84 (1.85)
Currently on antidepressant medication	25 (78.1%)
Currently on antipsychotic medication	7 (21.9%)
Currently in psychotherapy	8 (25%)
Currently on migraine medication	11 (34.4%)

**Table 2:** Descriptive statistics for outcome measures across timepoints.

Item	Baseline	After 10 Sessions	At 3 Months
Migraine QOL	51.56 (15.74)	72.75 (9.14)	76.88 (9.94)
HAMA	17.69 (3.16)	12.63 (3.01)	6.41 (1.74)
HAMD	15.41 (4.25)	9.56 (3.15)	7.90 (2.60)
MIDAS	16.22 (4.31)	11.22 (4.13)	n.a
HIT6	49.44 (12.58)	38.69 (9.83)	n.a

*n.a—Not assessed.*

**Table 3:** Changes across timepoints in anxiety and depressive symptoms, QoL, headache severity and disability.

Variable	Timepoint	Vs Timepoint	Mean Difference	Std Error	*p*-Value	95%CI
HAMA	Baseline	After 10 ses	4.838	0.507	<0.001	3.564, 6.112
		After 3 months	11.027	0.638	<0.001	9.424, 12.630
	After 10 ses	After 3 months	6.189	0.585	<0.001	4.721, 7.658
HAMD	Baseline	After 10 ses	5.778	0.384	<0.001	4.811, 6.744
		After 3 months	7.639	0.666	<0.001	5.965, 9.313
	After 10 ses	After 3 months	1.861	0.490	0.002 *	0.629, 3.093
Migraine-QoL	Baseline	After 10 ses	−21.459	1.909	<0.001	−26.253, −16.666
		After 3 months	−25.405	2.216	<0.001	−30.971, −19.840
	After 10 ses	After 3 months	−3.946	0.954	0.001	−6.341, −1.551
HIT-6	Baseline	After 10 ses	10.595	1.079	<0.001	8.406, 12.783
MIDAS	Baseline	After 10 ses	5.00	0.435	<0.001	4.112, 5.888

* After Bonferroni correction *p* = 0.006; all other comparisons remained at *p* < 0.001.

**Table 4:** RM-ANOVA and paired *t*-test results with corresponding effect size measures.

	df	F	*p*-Value	Partial Eta Squared (η^2^p)
HAMA	2, 62	181.984	<0.001	0.835
HAMD	1.43, 42.78	96.997	<0.001	0.764 **
Migraine-QoL	1.3, 40.34	94.157	<0.001	0.752 **
HIT-6	31	-	<0.001	1.61 *
MIDAS	31	-	<0.001	1.85 *

* Cohen’s d, paired *t*-test. ** Greenhouse–Geisser corrected degrees of freedom and F values reported due to violation of sphericity (HAMD: W = 0.597, *p* = 0.001, ε = 0.713; Migraine-QoL: W = 0.463, *p* < 0.001, ε = 0.651).

## Data Availability

The data presented in this study are available on request from the corresponding author and not publicly available due to privacy and ethical restrictions.

## Supplementary Materials

The following supporting information can be downloaded at: https://www.mdpi.com/article/10.3390/healthcare14091242/s1, STROBE Checklist.

## Abbreviations

The following abbreviations are used in this manuscript:

rTMS	Repetitive transcranial magnetic stimulation
iTBS	Intermittent theta burst stimulation
HAMA	Hamilton Anxiety Scale
HAMD	Hamilton Depression Scale
HIT-6	Headache Impact Test
MIDAS	Migraine Disability Assessment Test
QoL	Quality of life
DLPFC	Dorsolateral Prefrontal Cortex
RM-ANOVA	Repeated Measures—Analysis of Variance
