# Supplementary material for: Temporal Changes in Affective Symptoms, Headache Burden, and Quality of Life Following rTMS Treatment in Migraine: A Longitudinal Study
Source: Healthcare (Basel). 2026 May 4;14(9):1242. doi: 10.3390/healthcare14091242 (PMC13163773; doi:10.3390/healthcare14091242)
Supplement: Supplementary file 1 [file healthcare-14-01242-s001.zip › healthcare-4205567-supplementary.pdf]

**STROBE Checklist for healthcare-4205567**

| Section | Item No. | Recommendation | Page No. | Relevant text |
|---|---|---|---|---|
| Title and abstract | 1a | Indicate study design | 1 | The study design is explicitly stated as a one-arm longitudinal study in both the title and abstract. |
| | 1b | Provide balanced abstract | 1 | The abstract summarizes background, objective, design, sample size, outcomes, methods, and results with appropriate caution. |
| Introduction | 2 | Background/rationale | 3 | The introduction outlines migraine pathophysiology and rationale for rTMS and longitudinal assessment. |
| | 3 | Objectives | 3-4 | Objective: examine longitudinal changes in symptoms and quality of life. |
| Methods | 4 | Study design | 4 | One-arm longitudinal design described early in Methods. |
| | 5 | Setting | 4 | Outpatient neurology clinic; recruitment Aug 2023–Dec 2025. |
| | 6a | Participants | 4 | Eligibility, exclusion, and consecutive recruitment described; full follow-up. |
| | 6b | Matching | n/a | Not applicable. |
| | 7 | Variables | 4-5 | Outcomes (HAMA, HAMD, QoL, HIT-6, MIDAS) defined. |
| | 8 | Measurement | 4-5 | Measurement methods for all instruments described. |
| | 9 | Bias | 5-6 (and Limitations) | Bias sources discussed (confounding, no control group, etc.). |
| | 10 | Study size | 6 | Sample size justified using G*Power. |
| | 11 | Quantitative variables | 5-6 | Continuous variables analyzed using RM-ANOVA and t-tests. |
| | 12a | Statistical methods | 5-6 | Statistical methods described (RM-ANOVA, t-tests, effect sizes). |
| | 12b | Subgroups | 5-6 | No subgroup analyses performed. |

| | | | | |
|---|---|---|---|---|
| | 12c | Missing data | 4, 5 | No missing data present. |
| | 12d | Follow-up | 3 | No loss to follow-up. |
| | 12e | Sensitivity analyses | 6 | Bonferroni correction applied. |
| Results | 13a | Participants | 6 | All 32 participants included and completed follow-up. |
| | 13b | Non-participation | n/a | No attrition. |
| | 13c | Flow diagram | n/a | Not included. |
| | 14a | Descriptive data | 6-7 | Participant characteristics in Table 1. |
| | 14b | Missing data | 5, 6 | No missing data. |
| | 14c | Follow-up time | 7 | Follow-up duration 3 months. |
| | 15 | Outcome data | 6-8 | Outcome data reported in Tables 2–4. |
| | 16a | Main results | 6-8 | Mean differences, CI, p-values reported. |
| | 16b | Categorization | n/a | Not applicable. |
| | 16c | Absolute risk | n/a | Not applicable. |
| | 17 | Other analyses | 7 | Sensitivity analysis performed. |
| Discussion | 18 | Key results | 9-10 | Key findings summarized. |
| | 19 | Limitations | 10 | Comprehensive limitations discussed. |
| | 20 | Interpretation | 9-10 | Cautious interpretation; no causal claims. |
| | 21 | Generalisability | 9-10 | External validity discussed. |
| Other | 22 | Funding | Disclosure section | No external funding. |
